# Human papillomavirus infection in oral fluids of HIV-1-positive men:prevalence and risk factors

**DOI:** 10.1038/srep06592

**Published:** 2014-10-17

**Authors:** Karen Gaester, Luiz A. M. Fonseca, Olinda Luiz, Tatiane Assone, Adriele Souza Fontes, Fernando Costa, Alberto J. S. Duarte, Jorge Casseb

**Affiliations:** 1 Laboratory of Dermatology and Immunodeficiencies, Department of Dermatology, University of São Paulo Medical School, Brazil; 2 Institute of Tropical Medicine of São Paulo, São Paulo, SP, Brazil; 3 Department of Preventive Medicine, University of São Paulo Medical School, Brazil

## Abstract

Human papillomavirus is one of the most common sexually transmitted diseases worldwide. The natural history of oral HPV infection is unclear, and its risk factors have not been explored. Immunocompromised individuals, as exemplified by HIV patients, are at high risk for HPV-related diseases. The mean of this study is to determine the prevalence ofHPV in the oral tract of HIV-1-positive male subjects and its association with risk factors. A total of 283 oral wash samples from HIV-1-positive men were tested. The oral fluid samples were used for DNA extraction and conventional PCR amplification; HPV genotyping was performed by hybridization. HPV genotyping revealed that nine samples (3.5%) were positive for HPV DNA; the major high-risk HPV types identified were 51 and 66. Worldwide studies have shown a variable prevalence of oral HPV. The diversity of genotypes and the high prevalence of multiple infections in HIV-infected subjects can be better explained by the effects of HIV-induced immunosuppression. The most important risk factors are unprotected sexual intercourse, but other factors for this infection have been described elsewhere including smoking, age and HIV-positive serostatus. In this study, smoking was the most important risk factor for acquiring oral HPV in HIV-1-infected subjects in Brazil.

## Introduction

### Taxonomy

Human papillomavirus (HPV) is one of the most common agents of sexually transmitted diseases worlwide^1^. More than 180 HPV genotypes have been classified into different subgroups, with 40 types identified in the genital mucosa^2^,^3^.

### Human papillomavirus infection

The natural history of genital HPV infection in men is difficult to evaluate because it depends on the anatomical site, sample, sampling methods and HPV DNA detection assays^4^. The highest prevalence of HPV infection in males is found in anogenital sites and in men who have sex with men (MSM). The presence of HPV infection has also been associated with the acquisition of HIV infection^4^.

### Oral HPV infection

The oral HPV infection has not been explored^5^,^6^,^7^,^8^. The prevalence of oral HPV varies from 0 to 70% in normal oral mucosa, ranges from 0 to 85% in potentially malignant oral disorders and ranges from 0 to 100% in oral malignances. This variability is attributed to discrepancies such as sampling variation and limitations of the molecular techniques used in different studies^9^.

### Risk factors and HPV

Exposure is determined by risk factors common to most sexually transmitted infections^10^. Little is known about the factors contributing to oral HPV infection. Several studies have shown that the number of life-time sexual partners, frequency of sex, oral sex or other intimate skin-to-skin contact increase the chance of being infected by HPV^10^,^11^. Additionally, other risk factors have been reported to affect disease development related to HPV infection, including smoking, alcohol use and immunodeficiency^12^,^13^,^14^.

### Immunodeficiency and HPV infection

HIV infection increases susceptibility to opportunistic infections caused by viruses^15^,^16^. Thus, several studies have found an increased prevalence of HPV in individuals who are also HIV-infected^17^. Likewise, HIV infection can influence the natural history of HPV, by increasing the likelihood of a persistent infection and achieving greater virulence^15^.

To date, limited information is available regarding the natural history of oral HPV infectionand which risk factors are associated with it. Similarly, little is known about the association between HPV and HIV infections in men^18^. The aim of this study was to measure the prevalence of HPV in the oral tract of HIV-1-seropositive men and to evaluate major risk factors for transmission.

## Methods

### Study population

Two-hundred and eighty-three HIV-1-seropositive male patients referred to the HIV-Out Clinic ADEE3002, Dermatology Department of hospital das Clinicas, University of São Paulo medical school - HC-FMUSP and the Institute of Infectious Diseases “Emilio Ribas” from December 2011 to May 2013were included in the study. All patients appeared to have healthy oral mucosa. The mean age was 43.81 years (22–72 years). After signing an informed consent form, patients answered a questionnaire containing information on their social-behavioral characteristics. The study was approved by the respective ethical boards from the Institute of Tropical Medicine of São Paulo and the from Hospital das Clinicas of FMUSP.

### Sample collection

Oral washes were performed as follows. Patients wereinstructed to performance oral rinse with 10 ml of sterile saline solution (0,9% sodium chloride – Baxter International), and the rinses were submitted to sample collection. After collection, samples went through a process of washing and centrifugation. They were then aliquoted and stored at −20°C for later analysis.

### Precautions for contamination prevention

Strict procedures were followed to avoid false-positive reactions due to contamination. DNA extraction, reagent preparation and addition of sample DNA were carried out separately. Each area has its own dedicated equipment and every test included a negative control amplification containing distilled water without a DNA sample. All working surfaces were decontaminated with sodium hypochlorite and alcohol (70%), before and after use.

### Laboratory methods

Samples were subjected to a DNA extraction process using a commercially available kit (Illustra Tissue and Cells GenomicPrep Mini Spin kit, Easton Turnpike, Fairfield, USA) and, run according the manufacturer's instructions. After this procedure, the DNA was stored at −80°C for later analysis.

Beta-globin PCR was performed for all samples as a control for DNA quality. The protocol used was previously standardized in the laboratory where the research was conducted. PCR was performed in a final reaction volume of 50 ul, containing 5 ul of template DNA, 10 × PCR buffer, 50 mM of MgCl_2_, 10 mM of dNTPs, 5 U/ml of Taq DNA polymerase and 10 pmol of each primer. The PCR conditions were as follows: pre-heated at 95°C for 4 minutes followed by 35 cycles of 1 minute at 94°C, 1 minute at 55°C and 1 minute at 72°C, with a final extension of 7 minutes at 72°C.

HPV detection was carried out with a conventional PCR using the primers MY09/11. The protocol used was previously standardized in the laboratory where the research was conducted. PCR was performed in a final reaction volume of 50 ul, containing 5 ul of template DNA, 0.05 U/ul of Taq DNA polymerase, 4 mM of MgCl_2_, 0.4 mM of each dNTP, of PCR Mastermix(*Thermo Scientific*/Walthan - MA) and a final concentration 4 pmol of the MY09/11 primers. The PCR conditions were as follows: preheatedat 95°C for four minutes followed by 40 cycles of 1 minute at 94°C, 1 minute at 55°C and 1 minute at 72°C, with a final extension of 7 minutes at 72°C. The presence of HPV DNA was confirmed using electrophoresis on a 2% agarose gel containing ethidium bromide.

Finally, a commercially available kit was used for HPV genotyping(PapillomaStrip High + Low Risk − Operon Immune & molecular diagnostics − Cuarte de Huerva/Zaragoza − Spain). PapillomaStrip is a test based on the reverse blot technique that allows the genotyping of DNA samples and is capable of detecting 18 human papillomavirus subtypes of low-risk (subtypes 6, 11, 40, 42, 43, 44, 54, 61, 62, 67, 70, 71, 72, 74, 81, 83, 84 and 91) and 19 human papillomavirus subtypes of medium-high risk (16, 18, 26, 31, 33, 35, 39, 45, 51, 52, 53, 56, 58, 59, 66, 68, 69, 73 and 82). The methods were carried out in accordance with the approved guidelines.

### Statistical data analysis

Descriptive statistics were performed with the statistics program SPSS 20.0 and displayed in tables showing frequencies and percentages. The bivariate analysis was performed with the epidemiological statistics of open code for public health – OpenEpi 3.01 software withoral HPV as the dependent variable and age, smoking and alcohol use status, use of condom in sexual relations, practice of oral sex, HIV viral load, CD4 count, and use of HAART, as independent variables. Variables attaining a p value lower than 0.05 on the bivariate analysis were entered in a multivariate logistic model in a forward stepwise fashion. The multivariate analysis was performed with the aid of Stata 12.0 statistical software(StataCorp. 4905 Lakeway Drive, College Station, TX).

## Results

Laboratory data and behavioral characteristics of all 283 patients were analyzed. All patients in this study were male and HIV-infected (data not shown), 83% were using HAART, 82.3% had T CD4 cell counts ≥ 350 cell/mm^3^ and 78.8% had undetected HIV viral loads (less than 50 copies).

Most of the patients had a college degree (45.2%), were single (53.4%), of white ethnicity (60.8%) and did not use drugs (62.5%). We also evaluated alcohol intake and tobacco use: 54.8% ingested some alcohol and 30.4% were current smokers, 3.9% of the patients had smoked for months and 26.5% had smoked for years.

In regards to sexual exposure, 41% reported having had sex with men in the last year, 73.9% reported using a condom with a casual partner and 53.4% reported having engaged in active oral sex. Furthermore, 54.3% had passive oral sex and 52.6% had active anal sex. Approximately 75.4% had no discharge, and 80.2% had no warts or wounds on the genital site.

HPV DNA was found in ten of the 283 samples (3.5%) (data not shown). The mean age of the HPV+ group was 42 years (26–56 years), 70% were using HAART, 80% had T CD4 cell counts ≥ 350 cells/mm^3^ and undetectable HIV viral loads. Most of the patients were single (80%), 50% had a college degree, most were of white ethnicity and did use drugs. Among these patients, 80% are smokers and 60% smoke more once a day (Table 1).

For sexual exposure, all of the HPV-positive patients reported having sex with men in the last year, with active anal sex and using a condom with casual partners, 80% had active oral sex, 40% had passive oral sex, 80% had no discharge or wounds and 90% had no warts at the genital site.

HPV DNA was genotyped, identify and classifythe subtypes in each sample (table 2). In the high-risk group, the most highly prevalent type was HPV-66 (60%) followedHPV-51 (40%). The HPV-58 type was found in three samples (30%) and the HPV-16, 56 and 69 types were each found once in different samples.

In the low-risk group, the HPV-6 and 83 types were found in two samples (20%) and the HPV- 44, 62, 67, 72 and 84 types were each found once in different samples. Three samples had multiple high-risk subtypes, and two samples had multiple low-risk subtype.

Variables such as sexual exposure, alcohol consumption, cigarette smoking, HIV serostatus, HIV viral load, CD4 cell count and HAART use were assessed as possible risk factors for oral HPV (table 3). The bivariate analysis was performed comparing the distribution of factors between HIV-infected men HPV-HIV coinfected men.

The variables that had significant results based on a p value less than 0.05 were included in the multivariate logistic regression analysis to determine which factors are a risk for oral HPV infection (table 4). The only factor significantly associated the detection of HPV DNA was smoking [OR (CI) = 10.04 (1.98–50.92), p < 0.01].

## Discussion

Worldwide studies have shown a variable prevalence of oral HPV in healthy individuals^19^. This variability may be due to factors such as the difference in the local mucosal immunity, saliva flow and immunoglobulins in the oral cavity, different methods of sample collection and analysis and propensity to micro trauma injuries^20^,^21^.

In HIV-infected subjects, oral HPV infection is more aggressive and more highly prevalent compared with a healthy population^16^,^19^,^22^. A comparative study including HIV-infected patients and healthy subjects foundthat 25.3% of HIV patients had an HPV infection in the oral tractcompared with 7.6% of healthy subjects^23^. In the United States, a study was conducted with 190 HIV-positive men, and found that 6.3% them had detectable HPV in their oral fluid samples^23^. On the other hand, a study conducted in theUnited States and Spain with 166 patients showed a prevalence of 21% of oral HPV in HIV-positive MSM individuals, of which 11.1% were low-risk types and 66.6% were high-risk types^24^. In our study, oral HPV infection was detected in 3.5% of HIV-positive patients, in accordance withboth previous results and with the variability found in the prevalence of oral HPV in several studies^9^.

The diversity of genotypes and the high prevalence of multiple infections in HIV-positive subjects can be better explained by the effects of HIV-induced immunosuppression^25^. Our results demonstrate that most HPV-infected patients had more than one high-risk type.

When comparing the anogenital and oral genotypes, some types could be found at both sites. Regarding the low-risk groups, HPV-6 and 11 are the most prevalent^26^. In our study, HPV-6 was found in 20% of the samples. In the high-risk group, HPV-16 and 18 were the most prevalent at the anogenital site followed by HPV-31, 33, 39, 45, 51, 52, 53, 58, 59, 66 and 73^27^. In our study, several high-risk anogenital types were found, especially HPV-66.

The most important classical risk factors are unprotected sexual intercourse and multiple sexual partners^20^. Several studies have suggested that a high number of lifetime sexual partners, and practices such as kissing, oral-penile sex and hand warts are predictive of oral HPV infection^20^,^28^.

Other factors for this infection have been described elsewhere, including smoking and HIV-positive serostatus^20^. Although some studies indicate that there is conflicting evidence on the relationship between cigarette smoking and HPV infection, several studies suggested that recent cigarette smoking was associated with a significantly increased risk of oral HPV^29^,^30^. According to Read and coworkers^14^, smoking causes oral epithelial thickening, periodontal disease and epithelial abrasion, thereby increasing the risk of HPV infection.

In this report, we examined the oral tract as a site associated with the presence of HPV infection. After controlling for potential confounding variables, the only risk factor remaining significantly associated with HPV infection in our patients was cigarette smoking (p < 0.001).

Studies suggest that HIV-infected individuals may have a higher risk of developing oropharyngeal cancer as well other HPV-associated cancers^31^,^32^,^33^. Some studies, indicate that highly active therapy (HAART) does not reduce the prevalence of oral HPV and that CD4 cell count increases the incidence, progression and persistence of HPV-induced lesion^16^,^34^.

In our study, there was no substantial difference between these factors, because the majority of our patients had undetectable HIV viral loads and their CD4 cell counts were greater than 350 cells/mm^3^.

We could not determine whether HPV in the oral cavity of our patients had caused any related disease, such as head and neck squamous cell carcinoma (SCCHN) associated with cigarette smoking. However, it is known that these HPV oral carriers are potential transmitters of the virus.

Finally, it is important to acknowledge that most HPV types found in our patients are high-risk types but that, those types are unfortunately not treated by the currently available marketed vaccines. As a highlight, more studies are needed to enable a better understanding of the association between oral HPV infection and its risk factors in HIV-positive people.

## Figures and Tables

**Table 1:** Distribution of risk factors relative to social habits and laboratory data among 273 HIV-1 positive patients according to their HPV status. Bivariate analysis performed with the OpenEpi statistic program (version 3.01) using the exact test

		HIV-infected subejcts(n = 283)		*p Value
*NA	HPV negative (n = 273) (%)	*HPV positive (n = 10) (%)		
***HIV Viral load > 50 copies/mL***				
Yes		58(21.2)	2(20)	0.641
No		215(78.7)	8(80)	
***T CD4 cells count < 350***				
Yes		48(17.6)	2(20)	0.553
No		224(82.4)	8(80)	
**HAART use**				
**Yes**		228(83.5)	7(70)	0.230
**No**		45(16.5)	3(30)	
***Alcohol Intake***				
Yes		149(54.6)	6(60)	0.498
No		124(45.4)	4(40)	
***Cigarette Smoking***				
Yes		78(28.6)	8(80)	0.001
No		195(71.4)	2(20)	
**Discharge from genital site**				
**Yes**		66(24.2)	3(30)	0.456
**No**		207(75.8)	7(70)	
**Wart from genital site**				
**Yes**		55(20.1)	1(10)	0.378
**No**		218(79.8)	9(90)	
**Wounds from genital site**				
**Yes**		46(16.8)	2(20)	0.529
**No**		227(83.1)	8(80)	

*NA: no answer.

*p value < 0.05 and confidential limits excluded. Null values (0.1 or [n]) are highlighted.

*HPV positive: the results oforal HPV genotyping were compared with genotypes that infect the anogenital site.

**Table 2:** HPV types* from 9 HPV positive patients

Sample ID	HPV High-Risk type	HPV Low-Risk type
**01**	66	62
**02**	16,66	06
**03**	51	----
**04**	56	44,83
**05**	51,66	72
**06**	58	----
**07**	51,58,66	84
**08**	51	06
**09**	66	----
**10**	58,66,69	67,83

*Types identified through PapillomaStrip high and low risk (Zaragoza, Spain).

**Table 3:** Distribution of risk factors relative to sexual intercourse among 273 HIV-1 positive according to their HPV status. Bivariate analysis performed with the OpenEpi statistic program (version 3.01) using the Fisher exact test

		HIV (n = 283)		*p Value
*NA (%)	HPV negative (n = 273) (%)	*HPV positive (n = 10) (%)		
***Sexual relationship last year***	10 (3.7)			
No sexual relationship		42 (15.4)	0 (0)	0.216
With women		94 (34.4)	0 (0)	0.03
With men		106 (38.8)	10 (100)	0.0001
With both, women and men		21 (7.7)	0 (0)	0.417
***Condom with casual partner***	10 (4.1)			
Yes		199 (72.9)	10 (100)	0.000001
No		64 (23.4)	0 (0)	
***Condom with steady partner***	15 (5.5)			
Yes		197 (72.2)	4 (40)	0.302
No		77 (31.7)	6 (60)	
***Active oral sex***				
Yes		143(52.4)	8 (80)	0.07
No		130(47.6)	2(20)	
***Passive oral sex***				
Yes		158 (57.9)	10(100)	0.004
No		115 (42.1)	0(0)	
***Active anal sex***				
Yes		139 (50.9)	10(100)	0.001
No		134(40.1)	0(0)	
***Passive anal sex***				
Yes		103(42.4)	4(40)	0.563
No		170(62.3)	6(60)	

*NA: no answer.

*p value > 0.05 and confidential limits excluded. Null values (0.1 or [n]) are highlighted.

*HPV positive: the results of oral HPV genotyping were compared with genotypes that infect the anogenital site.

**Table 4:** Factors associated with oral HPV entered on the multivariate logistic regression analysis using the STATA program

Oral HPV	P	OddsRatio	CI 95%	
Alcohol use	0.338	0.99	0.94	1.03
Smoking	0.01	10.04	1.98	50.92
Condom with casual partner	0.324	1.15	1.05	1.26
Sex relation with men	0.22	0.91	0.86	0.96
Passive Oral sex	0.331	0.94	0.90	0.97
